# Implications of a Multi-Step Trigger of Retinal Regeneration in the Adult Newt

**DOI:** 10.3390/biomedicines5020025

**Published:** 2017-05-20

**Authors:** Hirofumi Yasumuro, Keisuke Sakurai, Fubito Toyama, Fumiaki Maruo, Chikafumi Chiba

**Affiliations:** 1 Graduate School of Life and Environmental Sciences, University of Tsukuba, Tennodai 1-1-1, Tsukuba 305-8572, Japan; s1330259@u.tsukuba.ac.jp; 2 Faculty of Life and Environmental Sciences, University of Tsukuba, Tennodai 1-1-1, Tsukuba 305-8572, Japan; sakurai@biol.tsukuba.ac.jp (K.S.); maru@biol.tsukuba.ac.jp (F.M.); 3 Graduate School of Engineering, Utsunomiya University, Yoto 7-1-2, Utsunomiya 321-8585, Japan; fubito@is.utsunomiya-u.ac.jp

**Keywords:** newt, retinal regeneration, retinal pigment epithelium, cell cycle, β-catenin, MEK, ERK

## Abstract

The newt is an amazing four-limbed vertebrate that can regenerate various body parts including the retina. In this animal, when the neural retina (NR) is removed from the eye by surgery (retinectomy), both the NR and the retinal pigment epithelium (RPE) eventually regenerate through the process of reprogramming and proliferation of RPE cells. Thus far, we have pursued the onset mechanism of adult newt retinal regeneration. In this study, using an in vitro system, we found that both mitogen-activated protein kinase (MAPK)/extracellular signal-regulated kinase (ERK) kinase (MEK)-ERK and β-catenin were involved in cell cycle re-entry of RPE cells. MEK-ERK signaling activity in RPE cells was strengthened by retinectomy, and nuclear translocation of β-catenin in RPE cells was induced by attenuation of cell–cell contact, which was promoted by incision of the RPE or its treatment with ethylene glycol tetraacetic acid (EGTA). EGTA is a Ca^2+^ chelator that disrupts cadherin-mediated cell–cell adhesion. Reinforcement of MEK-ERK signaling activity was a prerequisite for nuclear translocation of β-catenin. These results suggest that retinectomy followed by attenuation of cell–cell contact may trigger cell cycle re-entry of RPE cells. This study, together with our previous findings concerning the proliferation and multipotency of adult newt RPE cells, provides insight into the mechanism of the multi-step trigger in which the onset of retinal regeneration in the adult newt is rigorously controlled.

## 1. Introduction

The newt is a urodele amphibian that belongs to a group in the family *Salamandridae* [1]. This animal has an exceptional ability: it is able to regenerate, as an adult, an entire retina even when the eye suffers the loss of a neural retina (NR) after a traumatic injury ([2,3,4]; for review, see [5,6]). The retina of the newt, that is composed of the NR and the retinal pigment epithelium (RPE), is structurally similar to those of other vertebrates [2,3,4]. The primary origin of the regenerated retina in the adult newt is the RPE cell, which is a terminally differentiated cell type [2,3]. These cells form a monolayer cell sheet on the back of the NR, and support its physiological function in the intact eye [7]. When the NR is removed from the eye by surgery (retinectomy), the newt regenerates the retina as follows: (1) RPE cells lose their epithelial characteristics, detaching from each other and leaving the basement membrane (Bruch’s membrane); (2) single RPE cells, which express multipotency markers while entering the S-phase of the cell cycle, aggregate in the vitreous cavity; (3) these cells, named RPE stem cells (RPESCs), differentiate into two cell populations; (4) these cell populations construct two progenitor layers, namely the pro-NR and pro-RPE layers, in the correct polarity, while progressing to the M-phase and proliferation; (5) the pro-NR and pro-RPE layers eventually regenerate new functional NR and RPE, respectively (for more details, see [2,3]).

It is generally accepted that the regenerative process is triggered by injury. However, in the newt, the underlying mechanisms are still uncertain. Thus far, to address how retinal regeneration in the adult newt is triggered, we have investigated cellular events and signaling pathways that are involved in cell cycle re-entry of RPE cells [8,9,10] as well as their acquisition of multipotent properties [3,11]. Our studies have predicted that at least two elements are necessary for cell cycle re-entry of RPE cells: mitogen-activated protein kinase (MAPK)/extracellular signal-regulated kinase (ERK) kinase (MEK)-ERK intracellular signaling activity and release from inhibition mediated by cell–cell contact [9]. However, it is still to be studied how MEK-ERK signaling activity is regulated. We demonstrated in the previous study that MEK-ERK signaling activity was strengthened within 30 min after retinectomy [10]. However, a follow-up study is necessary to determine whether retinectomy is a causal event or not since in that study we could not exclude a possibility that surgical operation prior to retinectomy (incision into the sclera/choroid or removal of the lens) might have been responsible for it. In addition, it remains to be studied what signals are regulated by attenuation of cell–cell contact, and how these two elements are connected to each other.

In the present study, we addressed these issues using our original in vitro system in which we can carry out retinectomy in a dish and follow the behaviour of RPE cells under controlled conditions [9]. We finally found that MEK-ERK signaling activity, which was enhanced by retinectomy (the first step of the trigger), was a prerequisite for β-catenin signaling that was stimulated by attenuation of the cell–cell contact (the second step of the trigger). This study, together with our previous findings, provides insight into the controls that exist for the onset of retinal regeneration.

## 2. Materials and Methods

All methods were carried out in accordance with Regulations on the Handling of Animal Experiments in University of Tsukuba (RHAEUT). All experimental protocols were approved by the Committee for Animal Experiments in University of Tsukuba (CAEUT).

### 2.1. Animals, Retinectomy, and Collection of Eyeballs

Adult Japanese fire-belled newts, *Cynops pyrrhogaster* (total body-length: 9–11 cm), were purchased from a local supplier in Japan and housed in containers [3,12]. We used a total of 99 individuals regardless of their gender. In all experiments, animals were anesthetized with 0.1% FA 100 (4-allyl 2-methoexphenol; DS Pharma Animal Health, Osaka, Japan) before surgical operation or sacrifice [12]. Retinectomy and the collection of eyeballs were carried out as described [12]. In brief, for retinectomy, animals were placed under a dissecting microscope (SZ61; Olympus, Tokyo, Japan), the dorsal half of the eye was cut open along the corneal-scleral junction, and the NR was carefully removed together with the lens. After surgery, animals were allowed to recover and kept in moist containers at 22 °C until experiments were performed. To collect eyeballs, anesthetized animals were decapitated. Morphological stages of regenerating retinas were determined according to previously established criteria [2].

### 2.2. Preparation and Incubation of the Retina-Less Eye-Cup (RLEC)

RLECs were prepared as described in our earlier study [9]. In brief, the normal eyeball, which was placed cornea-side-up on a membrane filter (HAWPO 1300, Millipore, Billerica, MA, USA) and immersed in phosphate buffered saline (PBS: 137 mM NaCl, 2.7 mM KCl, 8.1 mM Na_2_HPO_4_, 1.5 mM KH_2_PO_4_, pH 7.4), was cut along the equator and its anterior half was carefully removed. The posterior half (eye-cup) was incubated in the same solution for 20–30 min and the NR was carefully removed to make a RLEC. The RLEC was incubated for up to 10 days in newt standard culture medium (NSCM), which is composed of 80% L-15 medium (pH 7.5; 41300-039, Thermo Fisher Scientific, Waltham, MA, USA), 1% fetal bovine serum (26140079, Lot 1024914, Thermo Fisher Scientific) and 1% penicillin-streptomycin (15140-122, Thermo Fisher Scientific, Waltham, MA, USA). The medium was refreshed on day 5 of incubation. Note that in the RLEC condition, the serum used here does not affect the first cell cycle entry of RPE cells [9].

In experiments to examine the effect of retinectomy on MEK-ERK signaling activity in 60 min, RLECs and eye-cups were incubated in 80% L-15 medium containing 1% penicillin-streptomycin (pH 7.5). In experiments to examine cell cycle re-entry of RPE cells, BrdU (5 μg/mL; B5002, Sigma-Aldrich, St. Louis, MO, USA) was added to NSCM. In experiments to examine the effects of cell–cell contact attenuation, RLECs were treated in ethylene glycol tetraacetic acid (EGTA) solution for 60 min immediately after removal of the NR. In this case, newt saline was used as the control. The EGTA solution contained 115 mM NaCl, 3.7 mM KCl, 10 mM EGTA (E-4378; Sigma-Aldrich, St. Louis, MO, USA), 18 mM d-glucose, 10 mM HEPES and 0.001% phenol red (pH 7.5). The newt saline contained 115 mM NaCl, 3.7 mM KCl, 3 mM CaCl_2_, 1 mM MgCl_2_, 18 mM d-glucose, 10 mM HEPES and 0.001% phenol red (pH 7.5). After EGTA or saline treatment, RLECs were rinsed twice in 80% L-15 medium for 15 min each, transferred into NSCM and incubated. Cell–cell attachment in the RPE recovered during incubation in NSCM. In experiments to examine the effects of signal inhibitors, a β-catenin signal inhibitor XAV939, which was dissolved in DMSO (dimethyl sulfoxide; D2650; Sigma-Aldrich, St. Louis, MO, USA) at 10 mM, and a MEK1/2-specific inhibitor U0126 (V1121, Promega, Fitchburg, WI, USA), which was dissolved in DMSO at 2 mM immediately before use, were administrated at a final concentration of 10 and 5 μM, respectively. For the mock control of XAV939, only the solvent DMSO was administered. For the mock control of U0126, an inactive analog of U0126, U0124 (662006, Millipore), was substituted for U0126.

### 2.3. Immunohistochemistry

#### 2.3.1. Antibodies

The primary antibodies used were rabbit polyclonal anti-phospho-ERK1/2 antibody (1:150; Phospho-p44/42 MAP kinase antibody, 9101S, Cell Signaling Technology, Danvers, MA, USA; [9,10]), rabbit polyclonal anti-N-cadherin antibody (1/200; ab12221, Abcam, Cambridge, UK; [4], mouse monoclonal anti-β-catenin antibody (1:1000; C7207, Sigma-Aldrich; Saint Louis, MO, USA, [13]) and mouse monoclonal anti-RPE65 antibody (1:1000; MAB5428, Millipore; Billerica, MA, USA, [2]). The secondary antibodies used were biotinylated goat anti-rabbit IgG antibody (1:400; BA-1000, Vector Laboratories, Burlingame, CA, USA), biotinylated goat anti-mouse IgG antibody (1:400; BA-9200, Vector Laboratories, Burlingame, CA, USA), Alexa 488-conjugated goat anti-rabbit IgG antibody (1:500; A-11034, Thermo Fisher Scientific, Waltham, MA, USA) and tetramethylrhodamine-conjugated goat anti-mouse IgG antibody (1:200; T2762, Life Technologies, Rockville, MD, USA).

#### 2.3.2. Tissue Preparation

For immunohistochemistry with tissue sections, normal and retinectomized eyeballs and RLECs were fixed in modified Zamboni’s solution for 5–6 h at 4 °C and cryosectioned at a thickness of ~20 μm [3]. The modified Zamboni’s solution contained 2% (*w*/*v*) paraformaldehyde and 0.2% (*w*/*v*) picric acid in PBS. For whole mount staining, RLECs were fixed in the same fixative for 3–4 h at 4 °C, except for those for BrdU immunostaining in which RLECs were fixed in 4% paraformaldehyde in PBS for 15 h at 4 °C.

#### 2.3.3. Immunofluorescence Labeling

Tissue sections and RLECs were immunostained as described previously by our group [3,9]. In brief, for immunofluorescence labeling, they were washed in PBS, 0.1% Triton X-100 in PBS, and PBS for 15 min each. The washed preparations were incubated with blocking solution for 2 h, and then incubated with primary antibody diluted in blocking solution for 15 h at 4 °C. They were washed and then incubated with secondary antibody diluted in blocking solution for 4 h. The blocking solution used here contained 2% normal goat serum (S-1000; Vector Laboratories, Burlingame, CA, USA), 5% bovine serum albumin (A3294-50G; Sigma-Aldrich, St. Louis, MO, USA) and 0.1% Triton X-100 in PBS.

#### 2.3.4. Immunoperoxidase Labeling

For immunoperoxidase labeling, tissue sections were pretreated in a sodium citrate buffer (10 mM sodium citrate, 0.05% Tween 20, pH 6.0) at 90 °C for 10 min [3]. The tissue sections and RLECs were washed, treated with 0.3% H_2_O_2_ in PBS for 20 min, washed, incubated in blocking solution mixed with Avidin D (1:50; Avidin/Biotin Blocking kit; SP-2001; Vector Laboratories, Burlingame, CA, USA) for 2 h, washed twice in PBS, and then incubated in primary antibody diluted with blocking solution mixed with biotin (1:50; Avidin/Biotin Blocking kit; SP-2001; Vector Laboratories, Burlingame, CA, USA) for 15 h at 4 °C. They were washed, incubated with biotinylated secondary antibody diluted in blocking solution for 4 h, washed, incubated in a mixture of Avidin and Biotin Complex (1:50 each; Vectastain ABC Elite kit; PK-6100; Vector Laboratories, Burlingame, CA, USA) for 2 h, washed, and then treated with DAB solution (DAB substrate kit; SK-4100; Vector Laboratories).

#### 2.3.5. Double Labeling

For double staining with β-catenin and RPE65 antibodies, tissue sections were processed for β-catenin immunoperoxidase labeling right up to ABC treatment. The sections were washed thoroughly, incubated with normal mouse IgG (1:200; 15381, Sigma-Aldrich, St. Louis, MO, USA) diluted in serum-less blocking solution for 2 h, washed thoroughly, and incubated with goat anti-mouse IgG Fab fragment (1:100; 115-007-003, Jackson ImmunoResearch, West Grove, PA, USA) diluted in serum-less blocking solution for 15 h at 4 °C. After washing thoroughly, the sections were processed for RPE65 immunofluorescence labeling. Finally, β-catenin immunoreactivity was visualized by ABC-DAB protocols. The serum-less blocking solution used here contained 5% bovine serum albumin (A3294, Sigma-Aldrich, St. Louis, MO, USA) and 0.2% TritonX-100 in PBS.

#### 2.3.6. Bleaching of Melanin Pigments and Nuclear Staining

After labeling, tissue sections were washed and occasionally bleached with 15% H_2_O_2_/1.5% sodium azide in PBS [3,9]. They were counterstained to visualize nuclei with either DAPI (4,6-diamidino-2-phenylindole; 1:50,000; D1306; Thermo Fisher Scientific, Waltham, MA, USA) or TO-PRO^®^-3 iodide (1:10,000; T3605; Thermo Fisher Scientific, Waltham, MA, USA), and finally mounted with 90% glycerol in PBS or with Vectashield mounting medium (H-1000; Vector Laboratories). For RLECs, RPE-choroid tissues were isolated under a dissecting microscope, placed RPE-side up on a glass slide and mounted under a cover slip with 90% glycerol in PBS. After the number of RPE cells were counted under a microscope (see below), the preparations were immersed in PBS so that the cover slip was detached and floated from the glass slide, allowing us to collect the RPE-choroid sheets. The recovered RPE-choroid tissues were then processed, similar to the tissue sections, for bleaching and nuclear staining followed by mounting.

### 2.4. Data Analysis

Transmitted light and fluorescence images of tissue sections and RPE sheets were taken by a charge-coupled device camera system (DP73; cellSens Standard 1.6; Olympus) attached to a fluorescence microscope (BX50; Olympus) or by a confocal microscope system (LSM510; LSM 5.0 Image Browser software; Carl Zeiss, Germany). For living PRE cells, images were taken using a digital camera (C-5060; Olympus) attached to a dissecting microscope (M165 FC; Leica Microsystems, Wetzlar, Germany).

For cell counting, the RPE in the RLEC was divided into two regions, “Edge” and “Center”, according to previously established criteria [9]: the Edge is defined as the 100 μm wide region along the incised margin of the RPE while the Center is the residual part of the RPE sheet (see Results). In tissue sections, RPE cells were identified by RPE65 immunoreactivity. The ratio of RPE cell nuclei with increased phosphorylated ERK1/2-immunoreactivity (pERK+) in the Center was obtained by dividing the number of RPE cell nuclei that were stained dark brown (the average luminance value <140; measured by a program in Photoshop CS5 Extended (Adobe Systems, San Jose, CA, USA)) by the number of all RPE cell nuclei (stained with DAPI). In this case, three sections from the same RLEC that contained the posterior pole of the eyeball and at a distance of 5 sections apart, were subjected to cell counting. The averaged value was used as a representative. In the case of RPE-choroid tissues (whole mount preparations), the total number of RPE cells was counted before bleaching (see above) by viewing their figures through Alexa 488 or DAPI filter sets on the fluorescence microscope [9,11]. In this condition, autofluorescence of the choroid tissue illuminates behind the RPE sheet and leaks through the boundary of RPE cells, allowing the hexagonal shape of RPE cells to be seen. β-Catenin immunoreactive (β-Catenin+) and BrdU immunoreactive (BrdU+) nuclei in the RPE sheet were counted after bleaching (see above) by focusing on nuclei (DAPI stained) on the uppermost cell layer. Each cell count was carried out by at least two people to increase accuracy and reliability.

In statistical analysis, to minimize the influence of individual animals, eyeballs of the same animal were used for both the control and the test. Data in the text are presented as the mean ± SE. In the graphs in which all data points were plotted, the values obtained from the same animal are shown by the same symbol and are connected by a dotted line which was drawn in the same color as the symbol. The statistical difference between the control and test was evaluated by a Sheffe’s pairwise comparison test following the Friedman test, except when data were homoscedastic, in which case a Student’s *t*-test (pairwise) was applied, by using Ekuseru-Toukei 2008 software (Social Survey Research Information, Tokyo, Japan).

Images were analyzed with software for image acquisition systems and in Photoshop CS5 Extended (Adobe Systems). Figures and panels were prepared using Photoshop CS5 Extended. Images, brightness, contrast and sharpness were adjusted according to the journal’s guidelines.

## 3. Results

### 3.1. MEK-ERK Signaling Activity in RPE Cells Is Strengthened by Retinectomy

We previously demonstrated that MEK-ERK signaling activity in RPE cells, which is detected at relatively low levels even in the intact eye, is strengthened within 30 min after retinectomy and gradually decreases to original levels by 6 h [10]. In that study, we suggested that this initial and temporal reinforcement of MEK-ERK signaling is required for cell cycle re-entry of RPE cells that takes place between 5 and 10 days after retinectomy [10]. In the present study, using an in vitro system, we confirmed that retinectomy is a causal event for this signal reinforcement.

**Figure 1:**
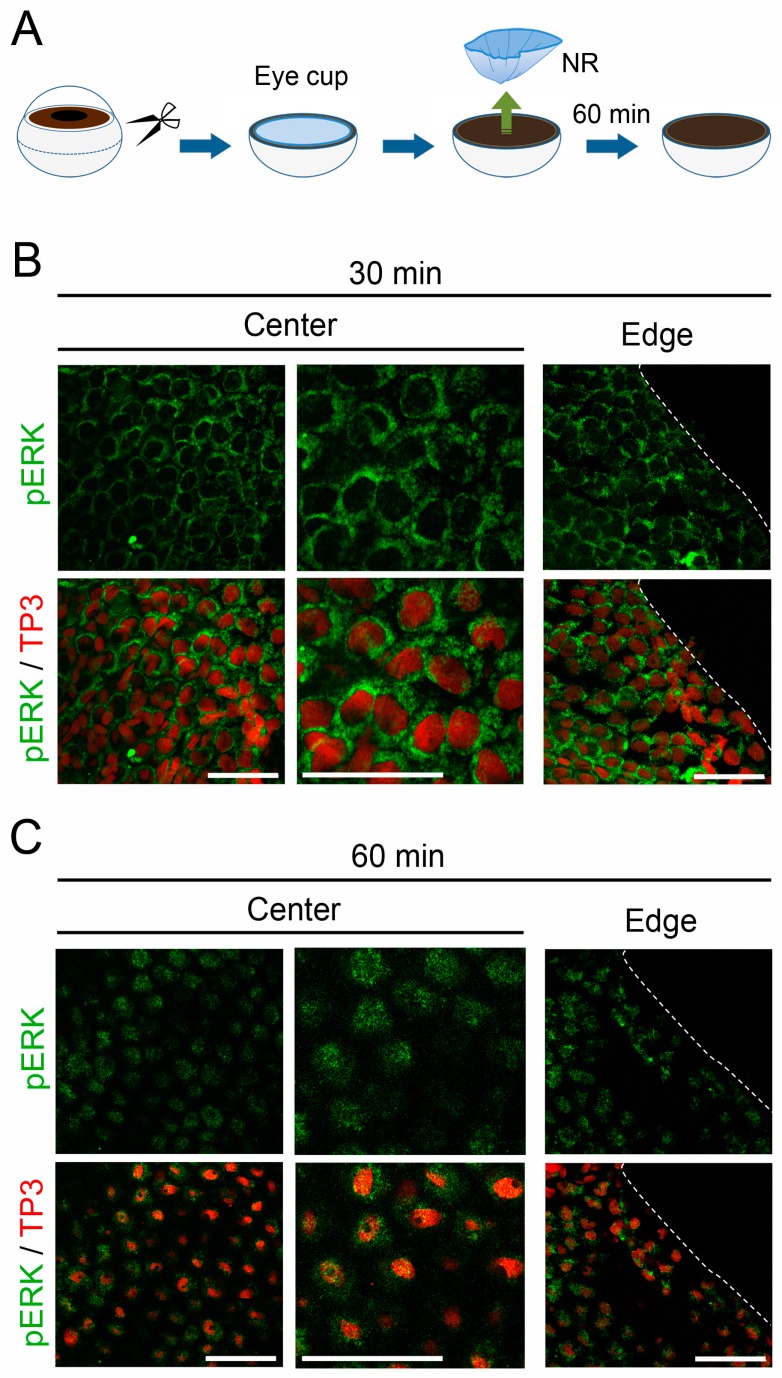
Retinectomy is a causal event for MEK-ERK signal reinforcement in retinal pigment epithelium (RPE) cells. (**A**) Schematic showing retinectomy in vitro; (**B**,**C**) Nuclear translocation of pERK in RPE cells after retinectomy in vitro. The Center and the Edge in the RPE sheet are shown. These are representative images (*n* = 3 each). Right-hand panels in the Center show magnified images of corresponding left-hand panels. TP3: nuclear stain by TO-PRO^®^-3 iodide. pERK immunoreactivity (green), which was observed in the cytoplasm of most RPE cells at 30 min after retinectomy; (**B**) became distributed to the nucleus (red) in the following 30 min; (**C**) Note that in these confocal microscopic images (optical slices), RPE cell nuclei at 60 min after retinectomy seemed to be smaller than those at 30 min, because the shape of the RPE cell nuclei, which was as flat as in intact cells at 30 min after retinectomy, changed into a spheroid within 60 min. Such a change in pERK immunoreactivity was observed simultaneously and uniformly throughout the RPE sheet. Scale = 100 μm.

If the surgical incision into the sclera/choroid along the equator of the eyeball is responsible for MEK-ERK signal reinforcement in RPE cells, the time course of the signal reinforcement and the distribution pattern of cells showing increased signaling activity would be different between the incised margin (i.e., the Edge) and the central area (i.e., the Center) of the RPE sheet. To examine this possibility, here we tried to visualize the activated ERK, or pERK, on a whole mount preparation by immunostaining (see Methods). When the eyeball was incised to make the posterior half of the eyeball (i.e., the eye cup) and the NR was removed from the eye cup in vitro, MEK-ERK signaling activity in RPE cells increased obviously within 30–60 min, as indicated by nuclear translocation of pERK (Figure 1). This was consistent with our previous observations in vivo [10], although progress was slightly slow in the current in vitro condition. Importantly, this change in MEK-ERK signaling activity took place simultaneously and uniformly throughout the RPE sheet. Therefore, the possibility that the surgical incision into the sclera/choroid might be a causal event for MEK-ERK signal reinforcement in RPE cells can be excluded.

**Figure 2:**
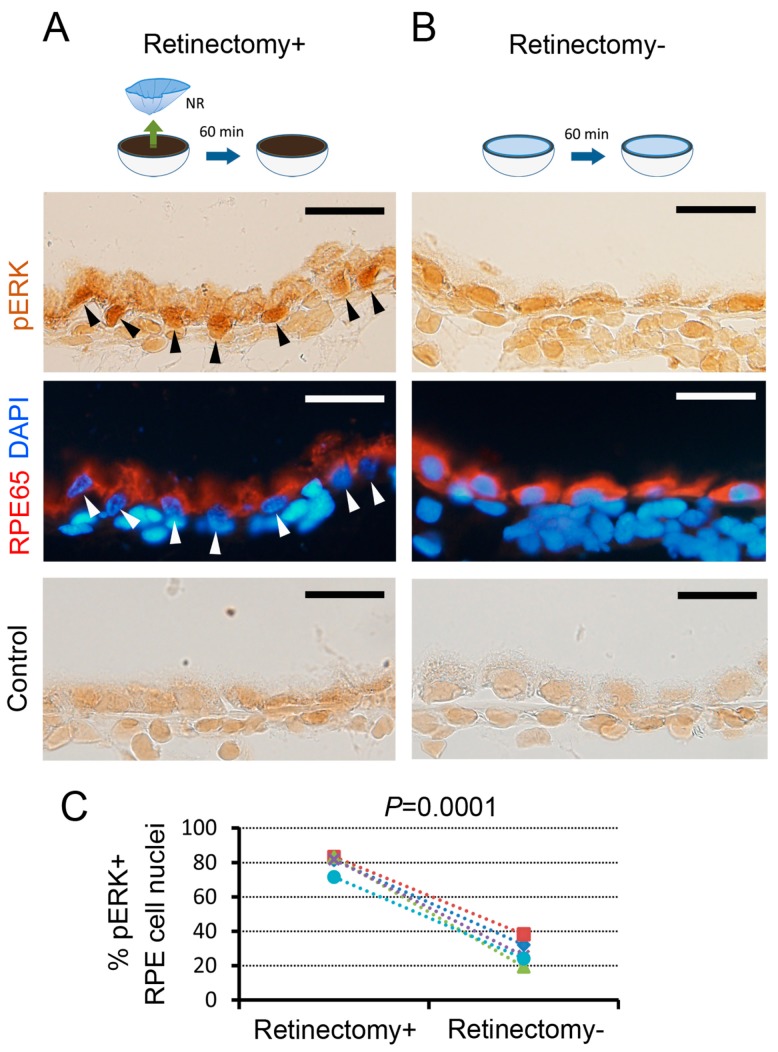
Effects of the neural retina (NR) on MEK-ERK signal reinforcement in RPE cells. (**A**,**B**) Representatives showing the effects of retinectomy on ERK activity in RPE cells (5 newts). Black and white arrowheads indicate pERK+ nuclei. Note that, for immunohistochemistry, the eye cups after 60 min incubation were fixed after removal of the NR. RPE cells were identified by RPE65 immunoreactivity (red). DAPI (blue): nuclei. To control immunoreactivity (lowermost panels), pERK antibody was replaced with a control IgG. Scale = 50 μm; (**C**) Proportion of pERK+ RPE cell nuclei in the Center. In retinectomy+, ~80% of RPE cell nuclei showed pERK+, whereas in retinectomy—the number of pERK+ RPE cell nuclei was decreased significantly to less than 40% (Student’s *t*-test, *p* = 0.0001, 5 newts).

Next, we examined whether the attachment of NR to the RPE could prevent MEK-ERK signal reinforcement in RPE cells (Figure 2). If the removal or lack of the anterior part of the eyeball including the lens is responsible for MEK-ERK signal reinforcement in RPE cells, this event would occur regardless of either the presence or absence of NR. When the NR was removed in vitro (retinectomy+), obvious nuclear translocation of pERK took place in 72–83% (80.3 ± 2.2%, *n* = 5) of RPE cells. On the other hand, when the eye cup in which the NR had been left intact was incubated in the same condition (retinectomy-), pERK+ RPE cells decreased to 19–38% (27.9 ± 3.3%, *n* = 5). This result indicates that retinectomy is essential for induction of MEK-ERK signal reinforcement in RPE cells.

These two results, together with our previous findings [9,10], suggest that retinectomy, but not preceding surgical operation, can be a trigger for cell cycle re-entry of RPE cells, or for retinal regeneration, via the temporal reinforcement of MEK-ERK signaling activity.

### 3.2. β-Catenin Signaling, Which Is Stimulated by Attenuation of Cell–Cell Contact, Promotes Cell Cycle Re-Entry of RPE Cells

Importantly, in in vitro conditions, RPE cells in the Center hardly enter the cell cycle, even though retinectomy is carried out ([9]; Figure S1). It has been suggested that cell–cell contact is responsible for this inhibition, because cell cycle re-entry of RPE cells was promoted along the incised margin of the RPE in the retinectomized eye cup (i.e., RLEC), and because it was stimulated either by removal of a piece of the RPE from the Center or by treatment of the RPE in the RLEC with EGTA solution, which attenuated cell–cell contact mediated by cadherin, a calcium-dependent cell adhesion molecule [9,14]. In addition, in amniotes, including humans, liberation from contact inhibition is an essential step for mature RPE cells to re-enter the cell cycle (for review, see [5]). Probably, in the RLEC in vitro conditions, factors which reduce cell–cell contact in the RPE in vivo are lacking [11].

In the present study, we examined signaling pathways that are activated by attenuation of cell–cell contact. For this, we first determined a condition of EGTA treatment of the RPE in the RLEC so that, unlike in our previous study [9], almost all of the RPE cells stayed on Bruch’s membrane over the period of incubation (10 days) (see Methods, and Figure S2). In this condition (i.e., EGTA treatment for 60 min), the proportion of cells which had entered the S-phase of the cell cycle in the Center within 10 days was on average about three times higher (range: 2.2–36.3%; 20.5 ± 5.2%, *n* = 6) than the control. However, the effect of EGTA treatment varied due to the individuality of animals (RPEs in 3 (50%) of 6 animals obviously responded). Thus, the EGTA treatment condition that we adopted in the present study provided a stimulus slightly above the threshold for cell cycle re-entry of RPE cells. It must be noted here that even without EGTA treatment (i.e., in the control condition), a small number of RPE cells in the Center re-entered the cell cycle because in rare occasions RPE cells in the Center sparsely fell out from Bruch’s membrane during retinectomy.

**Figure 3:**
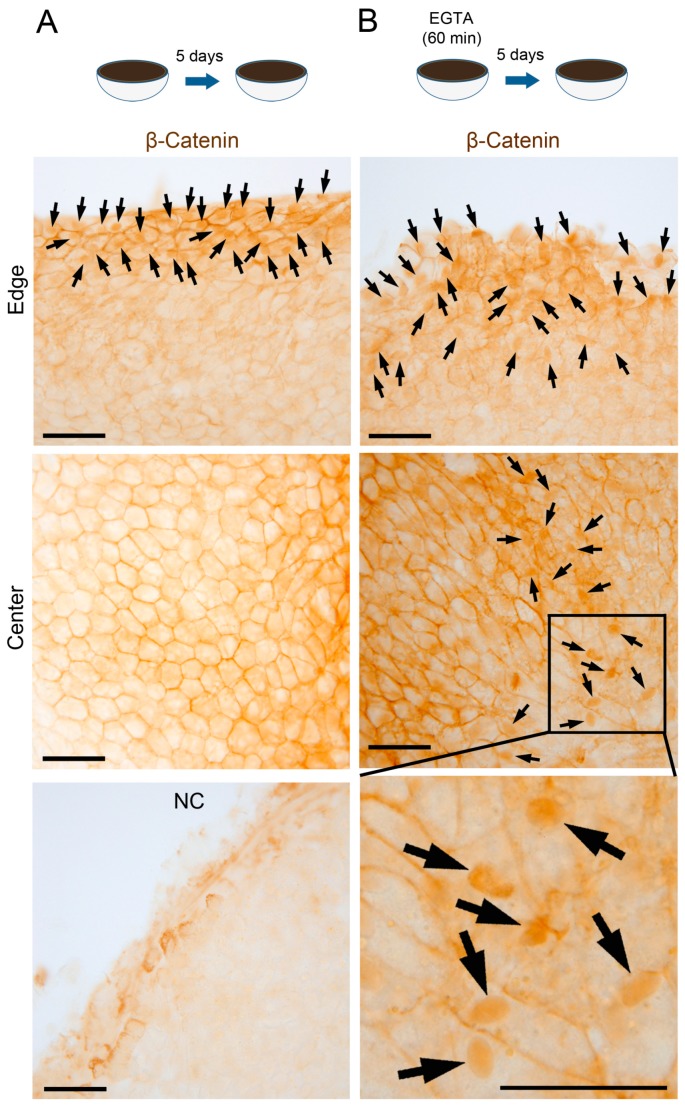
Effect of EGTA treatment on nuclear translocation of β-catenin in RPE cells. (**A**,**B**) Representative images showing β-catenin immunoreactivity in the RPE (bleached) on day 5 (5 newts). In the Edge, many nuclei showed intense immunoreactivity (arrows) in both conditions. In the Center, in the control condition; (**A**) intense immunoreactivity was localized along the cell membrane which was in contact with neighboring cells, while in the EGTA treatment; (**B**) it was observed in many nuclei (arrows) as well as along the cell membrane in the area where RPE cells changed their hexagonal shape to a rhombus or fusiform shape. NC: representative staining with control IgG as the primary antibody (*n* = 3). Note that weak nonspecific staining was observed in intercellular substances along the incised margin. Scale = 100 μm.

**Figure 4:**
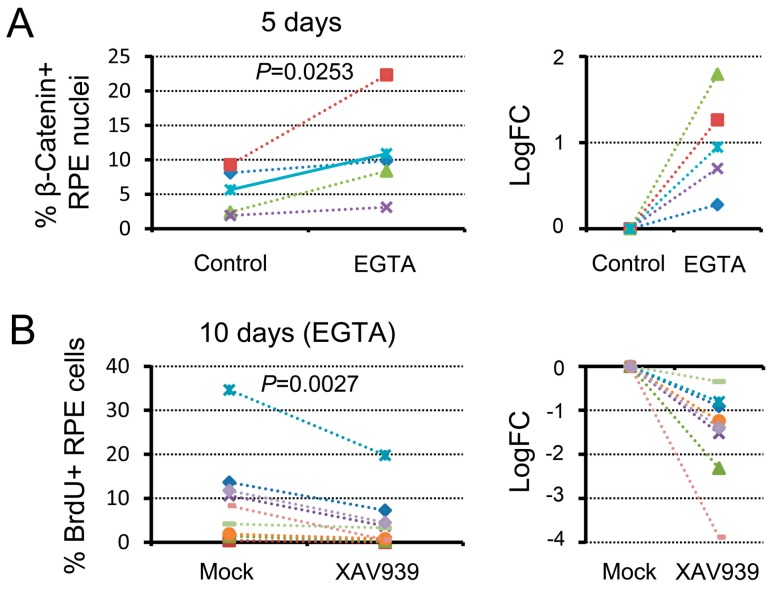
Attenuation of cell–cell contact promotes cell cycle re-entry of RPE cells via β-catenin signaling. (**A**) Differences in the ratio of β-catenin+ nuclei in the Center of the RPE on day 5 between normal saline (control) and EGTA treatment. The right hand graph shows the relative changes after EGTA treatment, and was plotted in log_2_ (fold change). The value increased significantly (Sheffe’s pairwise comparison test following the Friedman test, *p* = 0.0253) after EGTA treatment; (**B**) Effect of a β-catenin signaling inhibitor, XAV939, on cell cycle re-entry of RPE cells in the condition of EGTA treatment (9 newts). The left hand graph shows the proportion of BrdU+ cells in the Center of the RPE on day 10, and the right hand graph shows the relative changes in the presence of XAV939. The value decreased significantly (Sheffe’s pairwise comparison test following the Friedman test, *p* = 0.0027) in the presence of XAV939.

Under this set of conditions, we examined the effects of attenuation of cell–cell contact on the activation of β-catenin signaling. It is known that β-catenin, which associates with the intracellular domain of cadherin on the cell membrane, is released from cadherin and sends a signal to the nucleus, when cadherin-mediated cell–cell contact is disrupted by calcium depletion [15]. In the newt, RPE cells express N-cadherin ([12]; see below). When the RLEC was treated with EGTA solution for 60 min and then incubated in NSCM for 5 days, a proportion of β-catenin+ nuclei in the Center (range: 3.1–22.3%; 10.9 ± 3.2%, *n* = 5) increased significantly compared to the control without EGTA treatment (range: 1.9–9.3%; 5.5 ± 1.5%, *n* = 5) (Figure 3 and Figure 4A). In this study we chose this time point (day-5) because a majority of RPE cells have not re-entered the cell cycle in vivo [2,3] and in the Edge of the control [9]. In the control condition, β-catenin immunoreactivity was mostly localized on the cell membrane along the cell–cell contact region (Center in Figure 3A), as observed in either the intact RPE or the RPE immediately after retinectomy (see below). On the other hand, in the EGTA treatment condition, nuclear translocation of β-catenin was frequently observed in the area where RPE cells changed their hexagonal shape to a rhombus or fusiform shape (Center in Figure 3B). Probably the decrease of cell–cell adhesion allowed the cells to change their structure. The distribution pattern of such areas in the Center was different among RLECs. Note that those areas rarely appeared in the control condition.

We examined the effects of an inhibitor of β-catenin signaling, XAV939, on cell cycle re-entry of RPE cells in the EGTA treatment condition (Figure 4B). When the EGTA-treated RLEC was incubated in the presence of XAV939 for 10 days, the proportion of BrdU+ cells in the Center (range: 0–19.8%; 4.5 ± 2.1%, *n* = 9) decreased significantly compared to the mock control, which only contained solvent (range: 0.4–34.6%; 9.7 ± 3.5%, *n* = 9). Taken together, attenuation of cell–cell contact is likely to activate β-catenin signaling, which is involved in the cell cycle re-entry of RPE cells.

### 3.3. β-Catenin Signaling Is Activated within 3 Days after Retinectomy In Vivo

**Figure 5:**
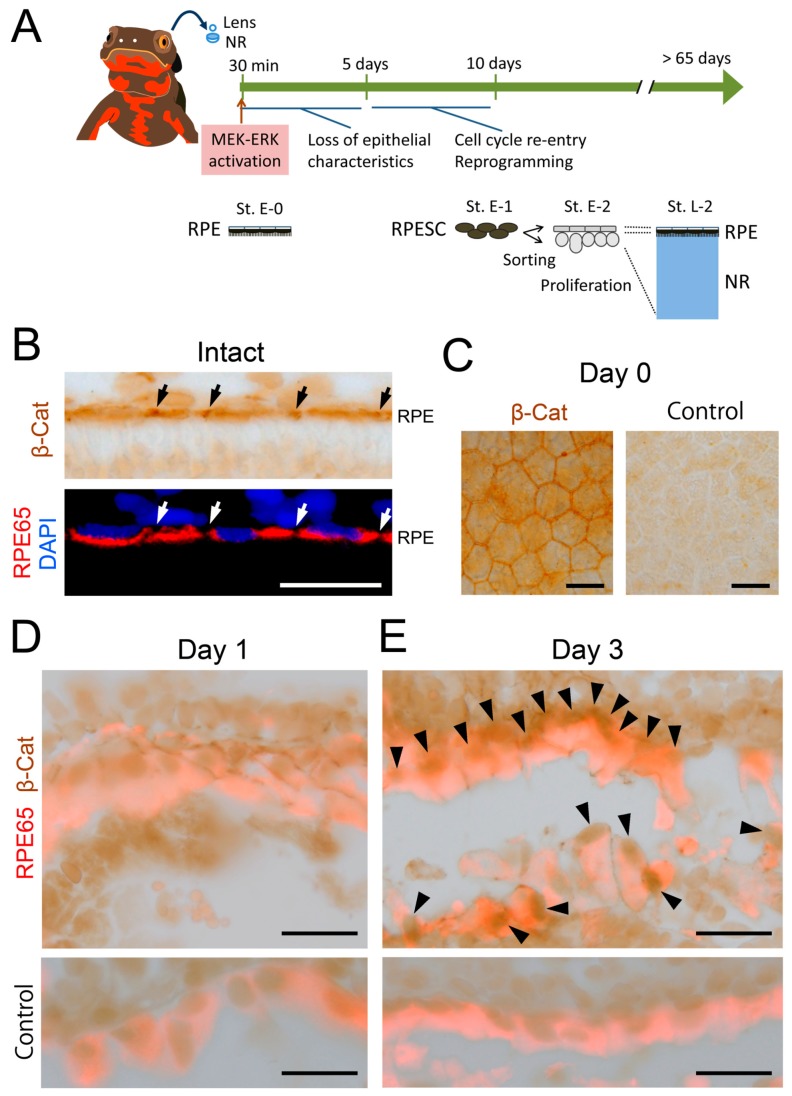
β-Catenin signaling is activated in 3 days after retinectomy in vivo. (**A**) Schematic showing events that take place during retinal regeneration; (**B**) Representative β-catenin immunoreactivity along the RPE layer in the intact eye (*n* = 3). The tissue was bleached. Intense immunoreactivity was observed in the region of cell–cell contact in the RPE (black and white arrows). Lower panel: RPE65 immunoreactivity merged with DAPI nuclear stain in the same region; (**C**) Representative β-catenin immunoreactivity in the RPE sheet of the eye immediately after retinectomy (day 0) (*n* = 3). Intense immunoreactivity was observed along the cell membrane which was in contact with neighboring cells. Right hand panel: representative staining with control IgG as the primary antibody (*n* = 3); (**D**) Representative β-catenin immunoreactivity in RPE cells at 1 day after retinectomy (*n* = 3). The tissue was double stained with RPE65 antibody and bleached. At this stage, immunoreactivity was not detected in the nuclei of RPE cells; (**E**) Representative β-catenin immunoreactivity in RPE cells/RPE-derived mesenchymal-like cells at 3 days after retinectomy (*n* = 3). Intense immunoreactivity was observed in the nuclei of RPE cells/RPE-derived mesenchymal-like cells (arrowheads), suggesting the activation of β-catenin signaling in this stage. Lower panels in (**D**) and (**E**): representative staining of the same stage of tissue using control IgG instead of β-catenin antibody (*n* = 3 each). Scale = 50 μm.

**Figure 6:**
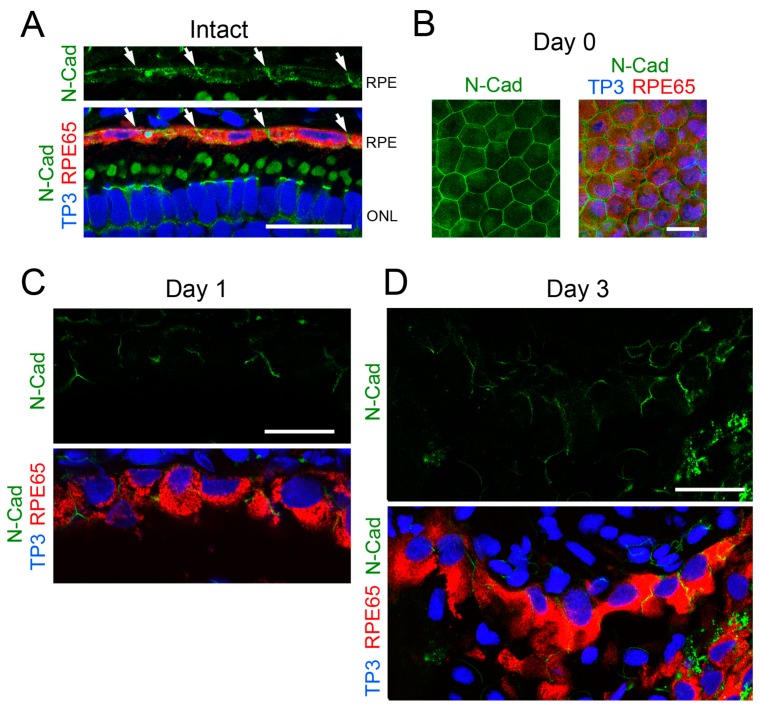
Change in the expression pattern of N-cadherin in RPE cells after retinectomy in vivo. (**A**) Representative N-cadherin immunoreactivity along the RPE layer in the intact eye (*n* = 3). Intense immunoreactivity was observed in the region of cell–cell contact in the RPE (arrows). Lower panel: merge of triple stain. RPE65 (red): RPE cells. TO-PRO-3 (TP3; blue): nuclei. ONL: outer nuclear layer; (**B**) Representative N-cadherin immunoreactivity in the RPE sheet of the eye immediately after retinectomy (day 0) (*n* = 3). Intense immunoreactivity was observed along the cell membrane which was in contact with neighboring cells. Right hand panel: merge of the triple stain; (**C**) Representative N-cadherin immunoreactivity in RPE cells at 1 day after retinectomy (*n* = 5). Lower panel: merge of the triple stain. At this stage, RPE cells still lined along Bruch’s membrane. N-cadherin immunoreactivity was recognized in the region of cell–cell contact; (**D**) Representative N-cadherin immunoreactivity in RPE cells/RPE-derived mesenchymal-like cells at 3 days after retinectomy (*n* = 5). Lower panel: merge of the triple stain. At this stage, cell–cell attachment in the RPE became loose but most of the cells still lay on Bruch’s membrane. In those cells, N-cadherin immunoreactivity was recognized along the cell membrane but in most cells the signal was low. Scale = 50 μm.

To confirm whether β-catenin signaling in RPE cells is activated in vivo, we examined changes in the subcellular localization of β-catenin at an early phase of retinal regeneration by immunohistochemistry (Figure 5). Figure 5A illustrates a summary of events that take place during retinal regeneration [3,10]. In both intact RPE cells and RPE cells immediately after retinectomy (day 0), β-catenin was mostly localized on the cell membrane along the region of cell–cell contact where N-cadherin was co-localized (compare Figure 5B,C with Figure 6A,B). After retinectomy, nuclear translocation of β-catenin was first recognized in RPE cells (71.5 ± 2.3%, *n* = 6) on the third day (Figure 5D,E), corresponding to the decrease of N-cadherin immunoreactivity along the cell membrane (Figure 6C,D). In this stage, cell–cell and cell–Bruch’s membrane attachment of RPE cells seemed to be loose, although most of the cells still stayed on Bruch’s membrane. These results suggest that β-catenin signaling in RPE cells is also activated in association with a decrease of their cell–cell contact in vivo, consistent with in vitro observations. As mentioned above, reinforcement of MEK-ERK signaling takes place within 30 min after retinectomy (Figure 5A). Hence, activation of β-catenin signaling seemed to take place later than reinforcement of MEK-ERK signaling.

### 3.4. MEK-ERK Signaling Is a Prerequisite for β-Catenin Signaling

**Figure 7:**
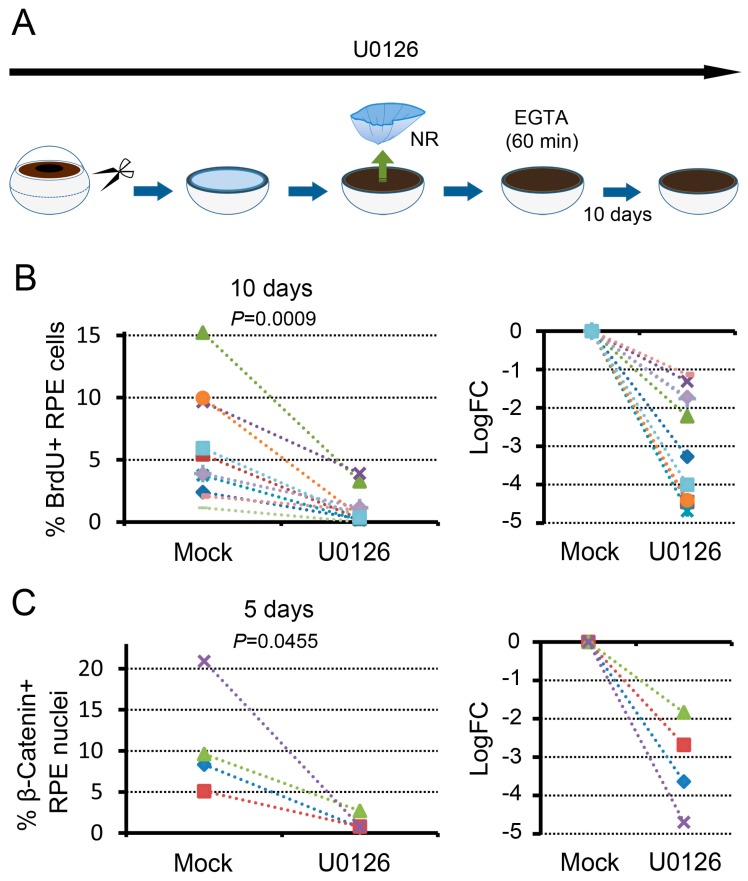
MEK-ERK signaling is a prerequisite for the promotion of cell cycle re-entry of RPE cells mediated by β-catenin signaling. (**A**) Schematic showing an experimental paradigm; (**B**) Effect of U0126 on cell cycle re-entry of RPE cells that was promoted by EGTA treatment (11 newts). The left hand graph shows the proportion of BrdU+ cells in the Center of the RPE on day 10, and the right hand graph shows the relative changes in the presence of U0126. The value was significantly lower (Sheffe’s pairwise comparison test following the Friedman test, *P* = 0.0009) in the presence of U0126; (**C**) Effect of U0126 on nuclear translocation of β-catenin in RPE cells that was promoted by EGTA treatment (4 newts). The left hand graph shows the proportion of β-catenin+ nuclei in the Center of the RPE on day 5, and the right hand graph shows the relative changes in the presence of U0126. The value decreased significantly (Sheffe’s pairwise comparison test following the Friedman test, *P* = 0.0455) in the presence of U0126.

We examined, using the in vitro system, the relationships between MEK-ERK signaling and β-catenin signaling, both of which are involved in cell cycle re-entry of RPE cells (Figure 7). For this, we administered a MEK inhibitor, U0126, from the time point when the eyeball was incised into the eye cup. In the presence of U0126, we carried out retinectomy, treated the resulting RLECs with EGTA solution for 60 min, and incubated them in NSCM for 10 days (Figure 7A). The concentration of U0126 was 5 μM which can inhibit the initial activation of ERK1/2 mediated by MEK1/2 up to ~50% [9]. In this set of conditions, the proportion of BrdU+ cells in the Center at 10 days (range: 0–3.9%; 1.1 ± 0.4%, *n* = 11) was significantly lower than the mock control with U0124 (range: 1.1–15.2%; 5.8 ± 1.3%, *n* = 11) (Figure 7B). This observation was consistent with our previous results [9]. In the same set of conditions, we examined β-catenin signaling after incubation for 5 days, and found that nuclear translocation of β-catenin was impacted by U0126: mock control, 5.1–20.9% (10.9 ± 3.4%), *n* = 4; U0126, 0.7–2.7% (1.2 ± 0.5%), *n* = 4 (Figure 7C). These results indicate that MEK-ERK signaling strengthened by retinectomy is a prerequisite for nuclear translocation of β-catenin or β-catenin signaling, which is stimulated by the attenuation of cell–cell contact.

## 4. Discussion

### 4.1. Adult Newt Retinal Regeneration Is Triggered through Multiple Steps

In the present study, we addressed the onset mechanism of retinal regeneration in the adult newt. We note that when the RLEC is placed in in vitro conditions, cells in a 100 μm-wide area along the incised margin (defined as the “Edge”) of the RPE spontaneously re-enter the cell cycle at high levels of probability, whereas in the other area (i.e., the Center), cells hardly re-enter the cell cycle due to cell–cell contact inhibition ([9]; Figure S1). In this condition, RPE cells in the Center survive for at least 10 days with no significant decrease in cell viability or apoptosis, and preserve their ability to re-enter the cell cycle as well as to re-express Pax6, an essential transcription factor for adult newt retinal regeneration [4], in the same time course as that observed during in vivo retinal regeneration [9,11]. Therefore, the Center of the RPE is an ideal subject to investigate factors or signaling pathways that trigger retinal regeneration.

**Figure 8:**
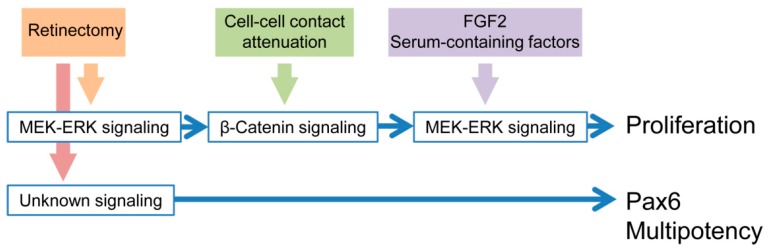
Conclusion. Retinal regeneration of the adult newt is triggered in a multi-step manner.

Focusing on the Center, we demonstrated that a combination of both retinectomy, which strengthened MEK-ERK signaling activity, and attenuation of cell–cell contact, which allowed nuclear translocation of β-catenin (i.e., β-catenin signaling), are necessary for cell cycle re-entry of RPE cells. Detachment from Bruch’s membrane seemed not to be required because in the current EGTA treatment condition, almost all of the RPE cells attached to Bruch’s membrane. We also showed that reinforcement of MEK-ERK signaling activity is a prerequisite for nuclear translocation of β-catenin. We obtained almost the same results in the Edge, except for the ratio of nuclear translocation of β-catenin, which was not affected by EGTA treatment (Figure S3). This can be explained by cell–cell contact of RPE cells in the Edge, which is impaired, as is their association with the NR during incision of the eyeball. In fact, the ratio of nuclear translocation of β-catenin in the Edge (range: 41.9–81.4%; 55.9 ± 6.8%, *n* = 5; Figure S3A) was higher than in the Center (range: 1.9–9.3%; 5.5 ± 1.5%, *n* = 5; Figure 4A). These results suggest that retinectomy followed by cell–cell contact attenuation triggers cell cycle re-entry of RPE cells. In other words, association with the NR and contact with neighboring cells prevent RPE cells from proceeding to the cell cycle, and thereby contribute to the preservation of their differentiated or physiological state. This mechanism explains well the fact that adult newt RPE cells can enter the S-phase of the cell cycle even in the absence of exogenous factors if they are isolated from the eye-cup [8]. However, for isolated RPE cells to proceed to the M-phase and the following proliferation stage, exogenous factors are necessary [8]. Candidates for those exogenous factors are FGF2 which activates MEK-ERK signaling, and other serum-containing factors that synergistically promote the effect of FGF2 [8]. Taken together, our studies imply a multi-step trigger of retinal regeneration (Figure 8). In fact, during retinal regeneration in vivo, reinforcement of MEK-ERK signaling activity in RPE cells takes place within 30 min after retinectomy [10], nuclear translocation of β-catenin becomes apparent at ~3 days (Figure 5), cell cycle re-entry takes place between 5 and 10 days, and mitosis followed by proliferation begins in the following few days [3]. Thus far, we have not known why the newt RPE cells require a long period (5–10 days) to re-enter the cell cycle after reinforcement of MEK-ERK signaling activity. This may be explained by the necessity of the second-step trigger that connects these events. It is well-known that the factor Wnt, involved in many biological processes and diseases, can up-regulate nuclear translocation of β-catenin [16]. However, a hypothesis that canonical Wnt/β-catenin signaling may promote cell cycle re-entry of adult newt RPE cells was not supported in our previous study [9].

We note here that double labeling of β-catenin and BrdU was not shown in the present study because of technical difficulties due to incompatibility of fixation conditions and usage of the primary antibodies produced in the same species (mouse in this case). In addition, it was difficult to carry out western blot analysis to confirm translocation of β-catenin because the proportion of RPE cells with β-catenin+ nucleus in the center was less than 25% in the current EGTA treatment condition (see Figure 4A). It is necessary to try other techniques such as reporter assay in the future. On the other hand, as to MEK-ERK signaling activity, we confirmed its temporal reinforcement by western blot analysis in our previous studies [9,10].

### 4.2. Mechanisms Underlying the First-Step Trigger

It remains to be studied how retinectomy stimulates MEK-ERK signaling in RPE cells, and how RPE cells lose their epithelial characteristics to relieve themselves from cell–cell contact inhibition in vivo. Our recent studies revealed that retinal regeneration in the adult newt is a homologous system to the epithelial to mesenchymal transition (EMT) of RPE cells that is observed in human RPE-caused retinal disorders such as proliferative vitreoretinopathy (PVR) [3,4,5]. However, the mechanism of PVR, including signaling pathways underlying EMT, remains obscure [17,18,19]. In in vivo retinal detachment (RD) or PVR models in mammals, it has been documented that RD activates ERK in RPE cells in 15 min [20], though the underlying mechanisms are still unsolved. One possibility is that RPE cells are relieved from inhibition, which is mediated either by direct contact of RPE cells with the NR via photoreceptor outer segments, or by factors that are constantly released from the NR in the physiological state [19,21]. Another possibility is an excitatory factor which is released from the NR tissue, and/or from the RPE itself, when the NR is separated from the RPE [19,21]. In PVR, many factors, including cytokines and growth factors that can activate RPE cells, have been reported, such as PDGF, FGF, EGF, IGF, VEGF, HGF, TGF-β and thrombin [5,17,18,19,22]. Interestingly, these factors can activate the pathways that converge into the MEK-ERK module [5]. In addition, it is important to consider a possibility that RD may cause the production of reactive oxygen species (ROS) because MAPKs are also involved in oxidative stress-induced response in RPE cells [23,24]. In the future, considering the time course of the MEK-ERK signal augmentation, we need to determine the mechanism/factors for the first step of the trigger for RPE proliferation in the adult newt. Such findings would also contribute to an understanding of the initial step of PVR in humans. Our newt in vitro system is a useful model to pursue this objective.

### 4.3. Mechanisms Underlying the Second-Step Trigger

In mammals, signaling pathways and molecular networks underlying EMT of RPE cells have been studied in in vitro PVR models, in which an RPE sheet established in culture, such as the ARPE-19 in humans, is used, although the RPE lacks communication with the NR [17,18,19,22]. Currently, TGF-β, especially TGF-β2, is regarded as the most important player in the EMT of RPE cells, as well as in their proliferation [17,18,19,22]. TGF-β2 induces the loss of epithelial markers such as E/P-cadherin and zonula occludens-1 (ZO-1), both of which are involved in cell–cell contact, and concomitantly stimulates an increase of mesenchymal (myofibroblast) markers such as α-smooth muscle actin (α-SMA), vimentin, fibronectin and collagen type IV. In mammalian RPE cells, N-cadherin, which is in general a major cadherin like E/P-cadherin, undergoes up-regulation during EMT. The decrease of E/P-cadherin by TGF-β2 leads to the increase of free β-catenin in the cytoplasm, allowing β-catenin to translocate to the nucleus [22]. In the RPE cell nuclei, β-catenin interacts with T-cell-specific transcription factor (TCF) and promotes, together with TCF, the transcription of genes including *cyclin D1* and *c-Myc*, leading to the activation of cyclin-dependent kinases responsible for cell cycle progression through the G1-phase to the S-phase [22]. Disruption of cell–cell contact inhibition by EGTA is known to substitute for TGF-β2 through activation of β-catenin signaling, although the expression of mesenchymal markers is not always significantly promoted [14].

In the adult newt, it is currently difficult to address whether TGF-β participates in the disruption of cell–cell contact inhibition in RPE cells, or if it is a factor for the second step of the trigger for RPE proliferation, because analytical tools are very limited in this animal. However, TGF-β signaling would be a leading candidate to be pursued in the future. In the present study, we found that in the early phase of retinal regeneration, nuclear translocation of β-catenin in RPE cells was apparently correlated to the decrease in N-cadherin immunoreactivity which took place when cell–cell adhesion of RPE cells decreased. In the RLEC, we found that the disruption of cell–cell contact by EGTA stimulated nuclear translocation of β-catenin and promoted the progression of RPE cells to the S-phase. We note here that unlike the EMT of mammalian RPE cells, in the early phase of retinal regeneration of the adult newt, N-cadherin immunoreactivity in RPE cells did not disappear completely, and seemed to increase in RPE-derived mesenchymal-like cells at Stage E1 (Figure S4). In the future, it is necessary to examine the participation of E/P-cadherin in the disruption of cell–cell contact. Importantly, in the adult newt, RPE cells do not display EMT during retinal regeneration, although RPE cells lose their epithelial characteristics and proliferate [4]. RPE-derived mesenchymal-like cells do not express either α-SMA or vimentin (Figure S4), but acquire multipotency to regenerate both the RPE and the NR. However, interestingly, when the normal reprogramming of RPE cells upon retinectomy is hindered by knockdown of Pax6, RPE-derived cells finally differentiate into myofibroblast-like cells, expressing α-SMA, vimentin as well as N-cadherin [4]. These findings provide insight into the possibility that an EMT program, which could be operated by TGF-β signaling in mammalian RPE cells, might be the operational background of the normal RPE reprogramming process. In the newt, the EMT program, particularly a component for installing multipotency, might be controlled in an elaborate manner to ensure retinal regeneration [4].

### 4.4. Triggering Mechanisms for Multipotency

In terms of multipotency that adult newt RPE cells acquire at an early phase of retinal regeneration, it is unclear whether the triggering mechanism is common to that for cell cycle re-entry. Reprogramming RPE cells, i.e., RPESCs, newly express transcription factors Mitf and Pax6 as well as pluripotency factors such as Sox2, c-Myc and Klf4 [3]. As mentioned above, Pax6 is a key factor for RPESCs to direct their fate from myofibroblast-like cells to retinal regeneration [4]. Very interestingly, in the RLEC in vitro condition, Pax6 is spontaneously expressed in RPE cells upon retinectomy, independently of either MEK-ERK signaling activity or cell–cell contact inhibition [11]. Therefore, for Pax6 expression at least, we must assume that other signaling pathways are associated with retinectomy (Figure 8).

## 5. Conclusions

In conclusion, the onset of retinal regeneration in the adult newt is rigorously controlled in a multi-step trigger with at least four signaling pathways (Figure 8). However, these entities remain largely unknown. Further understanding of these signaling pathways and molecular networks will contribute to the control of retinal disorder and regeneration in humans, as well as to better understanding of the onset mechanism of body-part regeneration in the adult newt.

## Supplementary Materials

Supplementary materials can be found at www.mdpi.com/2227-9059/5/2/25/s1.
