# Supplementary material for: Implications of a Multi-Step Trigger of Retinal Regeneration in the Adult Newt
Source: Biomedicines. 2017 May 20;5(2):25. doi: 10.3390/biomedicines5020025 (PMC5489811; doi:10.3390/biomedicines5020025)
Supplement: Supplementary file 1 [file biomedicines-05-00025-s001.docx]

Supplementary Information


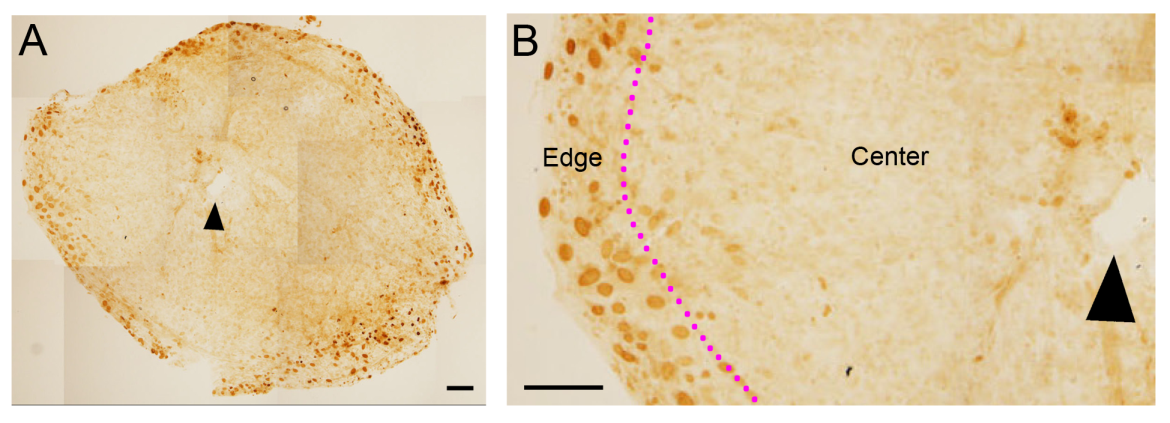


**Figure S1.** Cell cycle re-entry of RPE cells *in vitro*. When the RLEC was incubated in BrdU-containing NSCM for 10 days, most cell nuclei along the incised margin of the RPE were labeled with BrdU (BrdU+), whereas in the central area of the RPE, only a small number of nuclei were BrdU+ **(A)**. In the present study, we defined the ‘Edge’ as the 100 μm wide region along the incised margin of the RPE and the ‘Center’ as the residual part of the RPE **(B)**, as we did in a previous study [9]. The proportion of BrdU+ RPE cells was 40.5 ± 3.9% (range: 12.3-92.9%, *n* = 22) in the Edge, and was 5.6 ± 1.4% (range: 0-26.4%, *n* = 22) in the Center. The statistical difference in the proportion of BrdU+ RPE cells between these regions was significant (Sheffe’s pairwise comparison test following the Friedman test, *P*= 0.0000027, *n* = 22). Arrowhead: a hole which was made at the stump of the optic nerve when the RPE-choroid disc was isolated from a RLEC (see Methods). Scale = 100 μm.


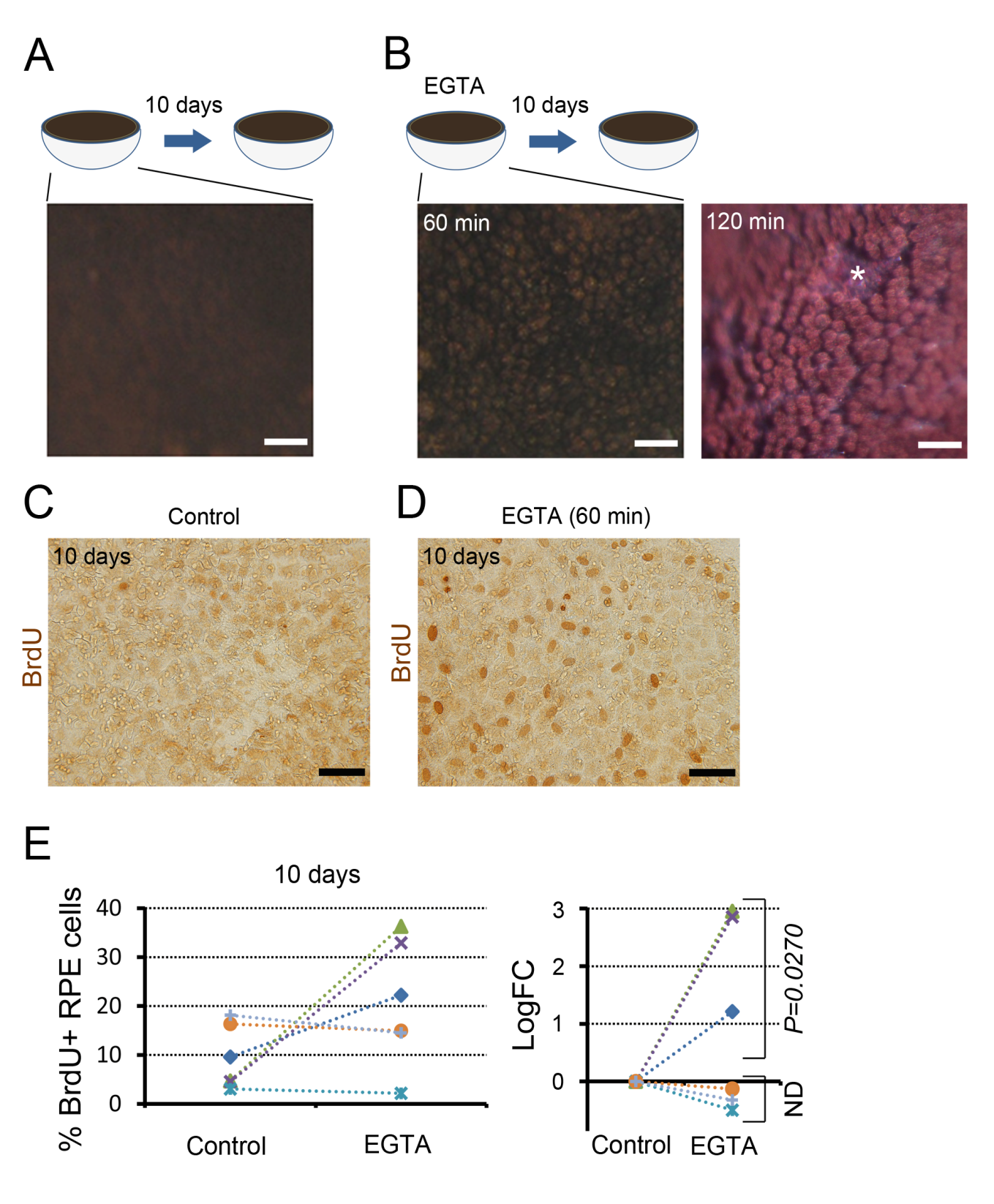


**Figure S2.** EGTA treatment of the RPE in the RLEC. We removed the NR from the eye cups, incubated the resulting RLECs in either normal newt saline or 10 mM EGTA solution for 60 min, and then incubated them in BrdU-containing NSCM for 10 days. **(A, B)** Representative images of the RPE after 60 min incubation in normal saline and EGTA solution, respectively (6 newts). **(B)** In EGTA treatment for 60 min (left-hand image), cell-cell attachment in the RPE decreased, allowing us to view the shape of cells under a dissecting microscope. The right-hand image shows an example of the RPE after longer incubation in EGTA solution. In this condition (120 min), the space between neighboring cells became more obvious but cells were sometimes dissociated from Bruch’s membrane (asterisk). **(C, D)** Sample images showing BrdU immunoreactivity in the RPE at 10 days after 60 min incubation in normal saline (control) and EGTA solution, respectively. In this case, as indicated by BrdU-labeled nuclei (brown), a large number of RPE cells re-entered the S-phase of the cell cycle in EGTA treatment **(D)**. The proportion of BrdU+ cells is shown in **(E)** as a brown X symbol. Scale = 100 μm. **(E)** Differences in the ratio of RPE cells which had re-entered the cell cycle in 10 days between normal saline (control) and EGTA treatment for 60 min. We counted nuclei labeled with BrdU in the RPE on day 10 and calculated the proportion of BrdU+ cells in the Center (left hand graph). The right hand graph shows the relative changes after EGTA treatment, and was plotted as log_2_ (fold change). Symbols linked by a dotted line show the data from the eyes of the same animal. We examined a total of 6 newts. In three of them, the values increased significantly after EGTA treatment (Student’s *t*-test, *P*=0.0270), although the other three did not show significant changes (ND). On average, the value was about three times (3.3 ± 1.4 times, n=6) higher in EGTA treatment. Note that RPE cells did not exhibit mitotic figures in 10 days as previously reported either *in vitro* [9] or *in vivo* [3].

**
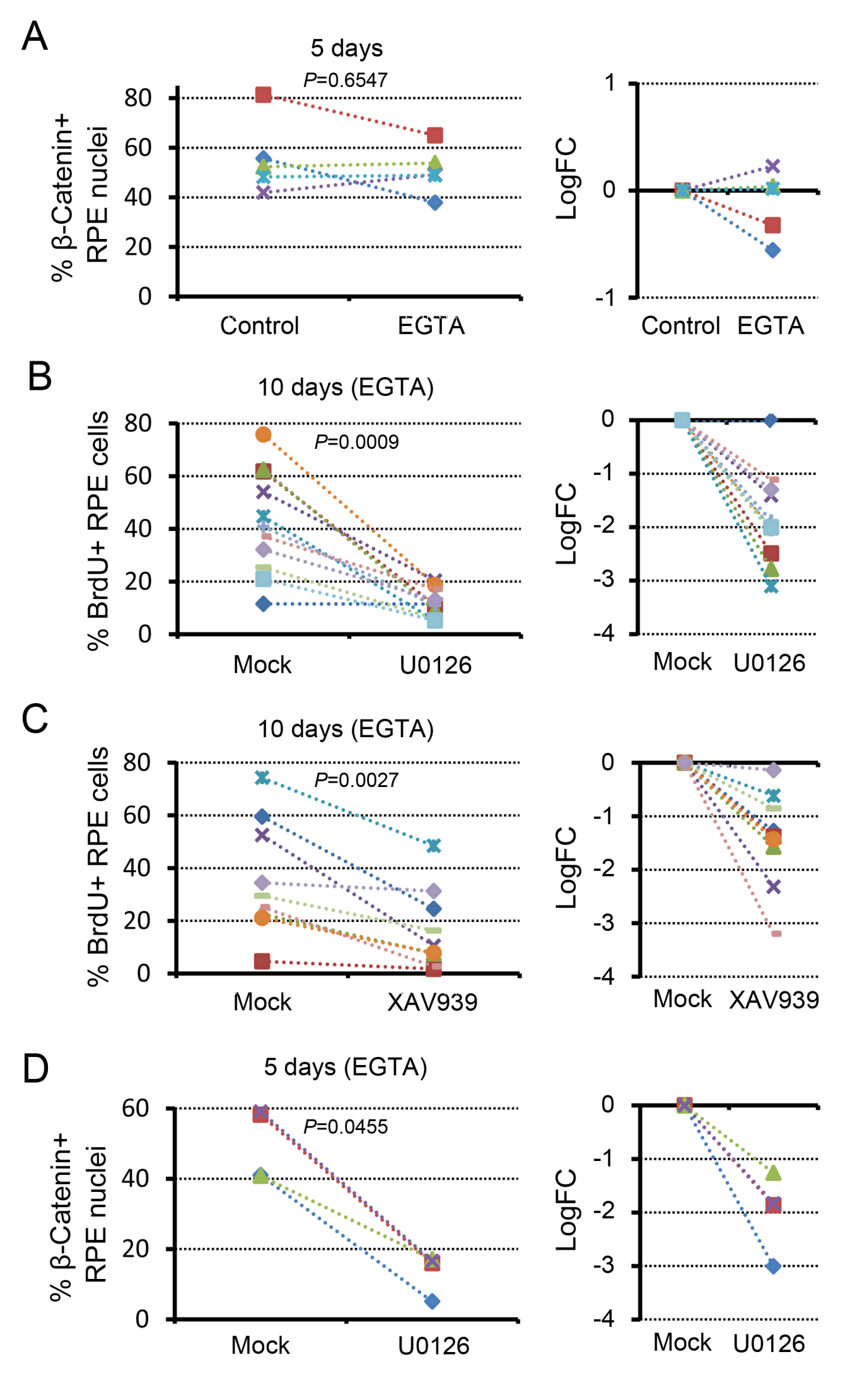
**

**Figure S3.** Results obtained from a 100 μm-wide area along the incised margin (defined as the ‘Edge’) of the RPE in the RLEC. **(A)** Effect of EGTA treatment on nuclear translocation of β-catenin in RPE cells. The data were obtained from the same samples (5 newts) used in Figure 4A. **(B)** Effect of a MEK inhibitor U0126 on cell cycle re-entry of RPE cells that was promoted by EGTA treatment. The data were obtained from the same samples (11 newts) used in Figure 7B. **(C)** Effect of a β-catenin signaling inhibitor XAV939 on cell cycle re-entry of RPE cells that was promoted by EGTA treatment. The data were obtained from the same samples (9 newts) used in Figure 4B. **(D)** Effect of U0126 on nuclear translocation of β-catenin in RPE cells that was promoted by EGTA treatment. The data were obtained from the same samples (4 newts) used in Figure 7C. Data are presented in the same manner as in corresponding data obtained from the Center. Statistical analysis was performed by Sheffe’s pairwise comparison test following the Friedman test.


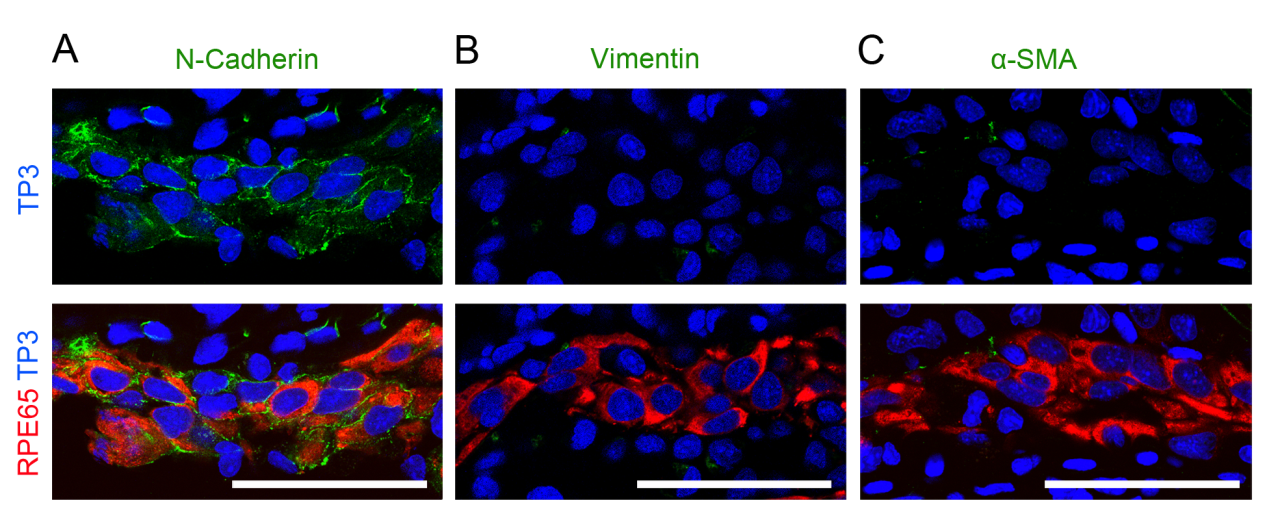


**Figure S4.** Immunolabelling of mesenchymal markers in RPE-derived mesenchymal-like cells at Stage E1 (10 days after retinectomy) in the adult newt. **(A)** Representative N-cadherin immunoreactivity (n=3). **(B)** Representative vimentin immunoreactivity (n=3). **(C)** Representative α-SMA immunoreactivity (n=3). N-Cadherin immunoreactivity was observed along the cell membrane of RPE-derived cells which had formed aggregates in the vitreous cavity, whereas immunoreactivities to other markers were not detected in RPE-derived cell. TP3 (blue): nuclei. RPE65 (red): RPE-derived cells. Scale = 100 μm.
